# Perceptions, Beliefs, and Experiences about the Menstrual Cycle and Menstruation among Young Women: A Qualitative Approach

**DOI:** 10.3390/healthcare12050560

**Published:** 2024-02-28

**Authors:** Alicia Botello-Hermosa, María González-Cano-Caballero, María Dolores Guerra-Martín, Carmen Flores Navarro-Pérez, Socorro Arnedillo-Sánchez

**Affiliations:** 1 Faculty of Nursing, Physiotherapy and Podiatry, University of Seville, 41009 Seville, Spain; abotello@us.es (A.B.-H.); marnedillo@us.es (S.A.-S.); 2 Faculty of Health Sciences, University of Granada, 18071 Granada, Spain; mariagcc@ugr.es; 3 Institute of Biomedicine of Seville (IBiS), 41013 Seville, Spain; 4 Midwifery Training Unit, Department of Materno-Fetal Medicine, Genetics and Reproduction, Hospital Universitario Virgen del Rocío, 41013 Seville, Spain

**Keywords:** dysmenorrhea, health knowledge, attitudes, practice, menstrual hygiene products, menstruation, qualitative research, taboo, women

## Abstract

The experience of menstruation is often associated with negative connotations and gender stereotypes, which results in making it invisible. This research aimed to explore the perceptions, beliefs, and knowledge of young Spanish women regarding the menstrual cycle and menstruation and their impact on their lives. The study delves into their understanding, menstrual management practices, the types of menstrual products employed, and their experiences related to menstrual health. Qualitative methodology was used with discussion groups as a data collection technique. The participants comprised 45 young Spanish women, aged between 18 and 23, hailing from both rural and urban areas. The majority were university students, with some engaged in part-time work, and one participant working full-time. While many experienced menstrual pain ranging from mild to debilitating, a normalization of this pain often led them to forego seeking specialist assistance. Disposable menstrual products (DMPs) are the most used by participants, despite limited awareness of their absorption capacity. Regarding reusable menstrual products (RMPs), menstrual cup users emphasized comfort but expressed a need for proper training. Negative menstruation experiences could evoke fear and difficulties, underscoring the importance of providing comprehensive menstrual health education encompassing both theoretical and practical components.

## 1. Introduction

Currently, in Spain, various studies indicate a lack of menstrual knowledge. The primary cause may be menstrual taboo, understood as the necessity to undergo the experience in secrecy and silence.

This concealment is evident in colloquial language, manifested through the prevalence of metaphors employed to describe the menstrual cycle [1], coupled with feelings of insecurity [2,3,4]. Consequently, a considerable number of adolescents and women currently encounter menstruation with a sense of embarrassment, limited understanding, and a tendency towards silence [5,6,7,8].

Furthermore, it is noteworthy that recent studies and systematic reviews provide evidence that a significant proportion of young women report experiencing varying levels of pain during menstruation, affecting between 45% and 95% of those in the menstruating age group [9]. Nevertheless, menstrual pain often remains concealed due to the factors mentioned earlier [10] and is frequently normalized, thereby leading to delayed diagnosis of certain pathological processes impacting women’s health [11].

Menstrual pain is categorized into two types: primary dysmenorrhea or secondary dysmenorrhea, contingent on its origin. Primary dysmenorrhea, the leading cause of pelvic pain among women, is not linked to any underlying disease. It is associated with the onset of bleeding, of short duration (lasting between 2 and 3 days), and typically does not reach a debilitating intensity. An increase in certain prostaglandins, leading to uterine contractions (cramps) and abdominal pain, is a contributing factor to its occurrence [12]. On the other hand, secondary dysmenorrhea refers to menstrual pain stemming from an underlying pathology, such as endometriosis or pelvic inflammatory disease [13].

Defined by the WHO “as a full physical, mental, and social well-being state and not merely as absence of diseases or malaise in relation to the menstrual cycle”, menstrual health has emerged as a burgeoning research area in the field of health, as evidenced by ongoing systematic reviews [14,15,16,17,18,19], given that more than 30 million women experience menstruation daily [17].

Recent studies underscore the need to comprehensively understand how women navigate and are affected by dysmenorrhea for its effective management. These factors are intricately tied to the sociocultural environment, and there is a dearth of information on these aspects among Spanish women [20,21]. This encompasses aspects such as their coping mechanisms and the types of menstrual products they utilise—whether disposable (e.g., pads, cotton tampons) or reusable (e.g., cups, underwear, towels).

Additionally, their knowledge of the subject warrants consideration, recognizing that traditional educational and health systems are losing relevance as sources of information. Instead, channels such as the Internet and social networks fill these gaps, albeit sometimes with content lacking objectivity or rigor [22].

Considering the gaps in understanding, this study is presented with the objective of delving into the perceptions of young Spanish women regarding their knowledge and beliefs about the menstrual cycle and menstruation. The study aims to explore how these aspects influence their lives, encompassing their understanding, menstrual management practices, the types of menstrual products used, and their overall experiences related to menstrual health.

## 2. Materials and Methods

### 2.1. Research Design

This is a qualitative study within the phenomenological–hermeneutic perspective, aiming to understand and explore the discourse of young Spanish women regarding beliefs and experiences related to the menstrual cycle and menstruation [23]. This design enabled us to delve into a specific topic in a particular context, seeking to comprehend the intricate world of subjective life experiences from the perspective of those who live them. The exploration is conducted in relation to their context and natural environment, considering holistic, dynamic, and individual dimensions [24]. The study adheres to the Standards for Reporting Qualitative Research (SRQR).

### 2.2. Participants, Sampling, and Recruitment

The study cohort comprised young women from Spain, aged between 18 and 23 years, residing in both rural and urban areas. The snowball sampling technique was employed for sampling. Initially, contact was established with 4 key informants, who facilitated the inclusion of additional participants meeting the inclusion criteria. In this context, the criteria were to be female and to be aged between 18 and 23 years.

### 2.3. Data Collection

For the purpose of data collection, focus groups were selected as the most appropriate method to fulfil the study objectives. A total of 4 focus groups were conducted between August and September 2022. The participants consisted of 45 women aged between 18 and 23 years, hailing from the provinces of Seville (the majority), Cádiz, Huelva, Badajoz, and Galicia, encompassing both rural and urban areas.

An effort was made to create balanced groups with minimal homogeneity elements to prevent situations that could impede collective discourse (such as differences in knowledge, power, and age). Additionally, the aim was to leverage common experiences while allowing for differences among participants, ensuring that excessive homogeneity did not hinder the groups.

Homogeneity criteria included age, rural/urban area, and educational level. Heterogeneity criteria encompassed age, ethnicity, rural or urban area, occupation, and province.

The criteria for considering an area as rural were based on towns with a population of less than 30,000, as stipulated by Spanish legislation [25]. The average number of participants per group was 11 women. The saturation criterion was used to conclude data collection.

An ad hoc script containing questions with the analysis categories already described was prepared to conduct the focus groups (Table 1).

The research objectives were outlined, and participants were requested to provide their informed consent before the commencement of the focus groups. Informed consent was duly obtained from all individuals involved in the study. The study adhered to the principles set forth in the Declaration of Helsinki and received approval from the Andalusian Biomedical Research Ethics Committee on 20 January 2017, with record number 14/2016.

For discourse analysis, interviews were transcribed verbatim following the collection of recordings, consistently prioritizing the privacy of the subjects. Each participant was designated with the letter P (participant) followed by a number according to their order of participation in the focus group. The data analysis was conducted post the literal transcriptions.

The assessment of qualitative data, encompassing words and texts, was executed through discourse analysis, involving segmentation by codes, formation of code families, and categories. This facilitated the exploration of the diversity of ideas collected during the data collection phase. A deductive approach was used for the creation of the categories; that is, the main categories were derived from the research question and the theoretical framework of reference. To ensure the trustworthiness of our methodology and enhance reliability, two researchers independently coded the transcriptions. The codes, categories, and assigned texts were triangulated with the rest of the team members.

This entire process was carried out using NVivo software, version 11, which serves as computational support in the analysis of qualitative data, such as interview transcriptions, field diaries, and observation records [26].

## 3. Results

### 3.1. Description of the Participants

The participant profile for the discussion groups is depicted in Table 2 based on the previously outlined homogeneity and heterogeneity criteria. The average age of participants across all four groups was 20.2 years.

### 3.2. Analysis Categories

The analysis categories were as follows: (1) Pain during menstruation. (2) Menstrual products. (3) Menstrual management. (4) Perceived knowledge about menstruation.

#### 3.2.1. Pain during Menstruation

One of the topics addressed was menstrual pain, where only five participants mentioned that they did not experience pain during their menstrual cycle and even considered themselves fortunate for not having it.

*“To be honest, I haven’t had pain since I started menstruating.”*

(P5, 20 years old.)

*“Most people experience pain, I’m the lucky one.”*

(P15, 19 years old.)

Other participants noted that it depends on the menstrual cycle, although the vast majority spoke of experiencing pain. At times, this pain was incapacitating and hindered their day-to-day activities; they categorized menstruation as dreadful, emphasizing that it was unusual for the cycle not to be painful. In addition to pain, they also referred to symptoms such as vomiting, headaches, dizziness, or drops in blood pressure.

*“The day before my period, my head always hurts a lot, and I start getting pains in the uterine area. Then on the day of my period, it depends; every other period hurts a lot.”*

(P12, 22 years old.)

*“In the last year, maybe 9 or 10 months, practically all my periods have been horrible, causing a lot of pain with many discomforts in the week before or even two weeks before. What used to be an occasional discomfort is now unusual not to feel much pain.”*

(P41, 20 years old.)

*“Many times, I’ve had to stay at home on weekends because, on top of the belly and kidney pain, I would get a fever from feeling so bad that I had to lie in bed with a warm blanket without moving because I was in terrible condition.”*

(P9, 21 years old.)

*“At first, it didn’t hurt much, and these last few years, it’s not every day, but there are days when the lower part of the abdomen hurts a lot. I feel like pinpricks, especially when I’m bleeding a lot, I feel that constant burden of blood, and I can’t eat or get up. Sometimes I want to get up, but I can’t because it feels like the world is falling apart from all the blood coming out.”*

(P5, 20 years old.)

*“My blood pressure drops, and many times, especially on the first day, the second, I get nausea, vomiting, it’s really hard for me.”*

(P22, 21 years old.)

The management of menstrual pain was also discussed, with the majority mentioning self-medication through the use of NSAIDs (nonsteroidal anti-inflammatory drugs). Some participants have had to go to the emergency department due to the pain they felt to receive medication there.

*“Now it’s true that the first or second day usually hurts, and I take a pill, an ibuprofen.”*

(P8, 20 years old)

*“I have also taken Enantyum© for my period, but it knocks you out; it puts you to sleep.”*

(P34, 21 years old.)

*“It’s terrible for me, the first 3 days, I have to constantly take a pill (Enantyum©) every 8 h because if not...”*

(P22, 21 years old.)

*“I’ve had pain to the point of waking up and starting to scream, telling my mother, ‘Mom, take me to the hospital because I’m dying,’ and going to the hospital almost fainting from the pain, literally bypassing triage, getting a drip, saline, and diazepam.”*

(P16, 22 years old.)

#### 3.2.2. Menstrual Products

Regarding menstrual products, the participants mainly made references about menstrual cups, although tampons and disposable pads seemed to be more commonly used. There was an awareness of emerging new menstrual health products.

*“I use menstrual pads and Tampax and the truth is that, right now, the brands are marketing a whole lot of things, not only menstrual cups but also knickers for menstruation”*

(P41, 20 years old.)

In relation to pads and tampons, these products seemed satisfactory and convenient to some participants who associated their usage with their menstrual flow. However, for others, fear of leaks and initial negative experiences reinforced the preference for tampons or the simultaneous use of both products.

*“I use menstrual pads and tampons because my period is not super abundant, Then I’m fine with menstrual pads and tampons.”*

(P7, 21 years old.)

*“Well, I’ve used tampons and menstrual pads since my first period, because the first day I had the period I was at school and I stained the chair, I panicked… In addition, I got really embarrassed, that’s why I’ve used tampons from the beginning.”*

(P6, 18 years old.)

Challenges faced when learning to use the menstrual cup were also addressed, basically regarding how to place it properly, finding the right size, comfort, and leaks. Once they learned how to use it, they started to perceive advantages like durability, although at times, they expressed fear it might came out during certain activities.

*“It was a little weird at the beginning because you have to learn how to put it, then it’s kind of you say “Phew” and you despair, but then, when you learn to use it and it also lasts 12 h because it’s made of silicone, you wake up and perhaps you don’t have to change it until the afternoon, you don’t need to worry about “Sometimes I need to alert in the classroom that it may came out, or if I’m doing some activity.”*

(P10, 20 years old.)

*“I started with cups last year and a whole lot better, to tell you the truth, because it’s like one fewer thing be concerned about, you don’t have to take an underwear change, simply that and that’s it, you don’t need to be counting the hours that you’re going to be outside to take one and when it is.”*

(P33, 21 years old.)

A participant revealed her experience with transitioning to a menstrual cup, finding that it was not enough for her excessive menstrual bleeding and going back to the use of pads, and finally using a combination of both menstrual cup and pads. She noted the better absorption quality of pads and now alters the use of these two menstrual products depending on the menstrual flow.

*“Both things in my case, yes, I tried some time ago, let’s see, I used more menstrual pads, then I started using cups, I found them very comfortable, but I bled a lot. And the cup was no good, then I went back to menstrual pads and now I’m using both, the days I bleed less I use cups and those I bleed more I use menstrual pads.”*

(P36, 20 years old.)

Unsuccessful transition to the use of the menstrual cup was also discussed, although it was seen as a good product due to its sustainability and its role as an alternative to pads in cases of allergy.

*“What I’ve always used are pads and Tampax, but the truth is that I bought a cup this year, but I couldn’t put it on well so that it’s comfortable, I have an allergy to menstrual pads and the cup is a whole lot more sustainable and, in the end, I would also be helping the environment.”*

(P40, 21 years old.)

In addition, fear about properly using menstrual products such as tampons was also observed in some testimonies; this idea was also repeated with menstrual cups.

*“I’ve always used pads until recently, well, until I had to use a tampon recently, but it’s just that I was very afraid of putting it on. It’s still hard for me, and the cup too.”*

(P3, 23 years old.)

#### 3.2.3. Menstrual Management and Costs

To the question “Have you ever had financial difficulties accessing menstrual products?”, most of the participants included in these focus groups stated that they had not; however, they stressed that they were expensive, that the most affordable ones were of poorer quality, and that they should be provided free of charge, especially to vulnerable groups.

*”I’ve never had difficulties, but I think that they should more economical because many people can’t afford them.”*

(P39, 19 years old.)

*“They’re expensive and the truth is that something should be done for those with few resources.”*

(P9, 21 years old.)

*“Now that I’ve moved out, I check the prices and choose the economical ones and I get a little mad because you can see that the material and all isn’t the same as other brands…“.*

(P1, 18 years old.)

#### 3.2.4. Knowledge about Menstruation

The study focused on the perception that young women have regarding their knowledge. Objective measurements of knowledge levels have not been undertaken.

When asked “What level of general knowledge do you believe you have about menstruation and the menstrual cycle?” the majority of participants asserted having high levels of knowledge. It is worth noting that these were predominantly university students, many of whom were enrolled in health sciences programs; hence, they may have perceived their knowledge levels as being high. It is important to emphasize that this perceived level of knowledge did not necessarily correlate with practical knowledge, particularly in terms of the use of menstrual products.

A distinction between theoretical and practical knowledge was made in the results.

*“They give you some insights in Biology at school, but they tell you the period is every 28 days, yet they don’t explain that some women don’t have it every 28 days, that there are people who are more irregular with their periods, and if it hurts a lot, you should go to the gynecologist because maybe you could have polycystic ovaries, which can be very painful, and that, nobody explains to you, they just say it’s normal.”*

(P13, 19 years old.)

This lack of practical knowledge translated into fears, as reflected in the menstrual products category.

## 4. Discussion

The principal findings obtained in the research were the normalization of menstrual pain, self-medication, and the need for practical information about menstrual products, as well as a growing interest in reusable products. While participants perceived their knowledge to be adequate, discourse indicated deficiencies in this regard. Participants were aware of the costliness of products and the decrease in quality of cheaper alternatives. Although they themselves might not have experienced menstrual poverty, they acknowledged its existence.

### 4.1. Pain

In our study, a significant number of participants reported experiencing some degree of pain during menstruation, an observation that aligns with the findings of a systematic review. In this review, it was highlighted that the prevalence of primary dysmenorrhea among participants included in the studies reached 71.5% [8]. It is noteworthy that none of our participants who experienced pain referred to having a menstrual disorder. This suggests that they may have internalized the perception that menstrual discomfort is normal. This finding is consistent with a previous study conducted among female university students in health sciences, where a substantial number of participants classified their menstruation as normal despite having an actual menstrual disorder [27].

Regarding the intensity of pain, our participants expressed that, in some instances, it was incapacitating, a phenomenon also documented in various studies. These investigations indicated that menstrual pain had impacted daily activities, including attendance at university [28]. It is worth noting that this year in Spain, the Organic Law 1/2023 has been enacted, allowing women with incapacitating dysmenorrhea to take leave from work for 3 to 5 days during their most painful periods [29].

The most common strategy for managing pain was self-medication; this aligned with the conclusions of several studies where participants with menstrual pain opted for this method to alleviate their symptoms [30]. As discussed, menstrual pain can significantly affect a person’s quality of life, making it difficult to carry out daily activities; therefore, recognizing and addressing menstrual pain is important to ensure the well-being and overall health of those experiencing it.

### 4.2. Menstrual Products

#### 4.2.1. Pads and Tampons

Participants predominantly use disposable menstrual products—pads and tampons—aligning with García Egea’s findings in the Spanish population [31]. Despite this prevalence, there is a growing interest in reusable menstrual products, reflected in an increased number of scientific publications on the subject.

Varied perspectives arise on the use of disposable pads. While they are considered comfortable for those with lower menstrual discharge, individuals with a heavier flow express concerns about potential leaks, causing discomfort due to past incidents or fear of their recurrence. This factor notably affects the social and academic aspects of young women’s lives, contributing to negative menstruation-related experiences that transcend national boundaries, regardless of economic development levels [17,32].

Some participants choose a combination of disposable pads with tampons or abstain from using them entirely due to these concerns. Additionally, there is a viewpoint among participants suggesting that disposable pads are considered unhygienic.

In general, the testimonials indicate that a significant number of participants attribute greater absorption capacity to tampons, viewing them as offering improved protection against leaks. Surprisingly, some women even connect tampon usage with the perception of shorter menstrual cycles. Notably, a study on health-related quality of life and menstrual products by Huang [33] reported more favorable outcomes for tampon users compared to those using disposable pads. This contradicts Garcia-Egea’s findings, where negative testimonials emerged regarding tampons and disposable menstrual products, citing concerns related to environmental impact and health [3].

In a minority of instances, a “fear” of using tampons is evident, especially among young women and particularly in young adolescents. A study in the USA with young women found that this fear originates from a lack of practical knowledge about menstrual products and their proper handling. The menstrual health education in schools, being predominantly science-focused, inadvertently contributes to the development of negative attitudes, embarrassment, and anxiety [34].

These findings underscore the significance of the quality and absorbency capacity of disposable menstrual products in shaping the lives and experiences of young women during menstruation. These products extend beyond mere menstrual products to become influential factors in young women’s menstruation experiences and the associated stigmatization. Consequently, it is imperative for them to undergo rigorous quality control measures within the industry, with their absorbency capacity rigorously tested and certified. Practical education concerning their usage should also be integrated into menstrual education programs.

#### 4.2.2. Reusable Menstrual Products

The discourses reveal a growing interest in reusable menstrual products, particularly menstrual cups, with no mention of menstrual underwear or reusable pads. A review on the utilization of reusable pads highlights their infrequent use [35]. García-Egea [30] found that 54.9% of women in Spain preferred reusable menstrual products, predominantly choosing menstrual cups (48.4%); this was especially the case among younger women and university attendees. Similarly, a recent Australian study reports that 37% of young women used some form of reusable menstrual product in their last menstruation, with 11% having experimented with them at least once [36]. Interestingly, menstrual underwear is the most utilized product (24%), followed by menstrual cups (17%) and reusable pads (5%). In the United Kingdom, menstrual cup usage stands at 20% [37].

Another profile includes young women who have experimented with menstrual cups but, for various reasons, choose not to continue using them. Some find menstrual cups inadequate for managing heavy flow and, as a result, prefer using disposable pads. Leaks occur in approximately 2–31% of cases when using menstrual cups; these leaks are primarily linked to conditions like metrorrhagia, changes in uterine anatomy, incorrect cup size, improper cup insertion, or failure to empty the cup promptly. Additionally, a notable concern associated with menstrual cup use is the challenge of finding cups of the right size [35,38].

Others do not find menstrual cups comfortable, potentially due to insertion difficulties, as noted by several authors who have highlighted vaginal/vulvar pain as a primary barrier to their use [31,35]. Factors like becoming familiar with the cup, practice, training, and peer support contribute to successful use [35,39].

An influential factor affecting the use of menstrual cups is having friends who have experienced positive outcomes with them [40,41,42]. Some participants learned about the product through friends or sisters and were even awaiting instructions on its use from them. In line with this, Jarrahi [43] recommends that health managers incorporate peer-teaching on menstrual hygiene. Practical guidance on size, insertion, and care is fundamental for young women to feel confident enough to initiate the use of menstrual cups. Despite the participants’ belief that tampons offer better absorption than disposable pads, pads, tampons, and menstrual cups share similar absorption capacities (20 mL–50 mL) [44]. Recently, a study on cup users found that the use of cups may alter the experience of menstruation by exposing users to sensory aspects and the unabsorbed nature of their menstrual blood, which elicits feelings that counteract the tendency to forget about menstruation [45]. These perspectives contrast with employing a passing technology, by means of which a women might temporarily pass as a non-bleeder [46].

These results confirm a growing trend and preference for reusable products among young women, reflecting their concern for the environment and sustainability. Consequently, there is a necessity for education on these products, focusing on practical aspects of their use and management.

### 4.3. Perception of Participants’ Knowledge about Menstruation

Unlike our study, in Barrington’s systematic review, many participants exhibited poor knowledge about menstruation, leading to distress in many of them [47]. A similar situation is observed in a study conducted in our country, where participants’ perceived knowledge is low [48], as reflected in the international plan presented in the United Kingdom [49].

The high perception of knowledge in our study may be attributed to the fact that many participants included were pursuing studies in the field of health sciences.

### 4.4. Menstrual Management and Costs

According to the analysis of testimonies, most participants reported no economic difficulties in purchasing menstrual products. However, they emphasized the high cost of these products and advocated for their provision free of charge, especially for vulnerable groups. In comparison to the Equality study, where a significant proportion (22%) faced economic challenges in acquiring menstrual products and nearly 40% encountered difficulties in obtaining their preferred products [30], our participants echoed similar concerns. Some mentioned resorting to lower-quality or less preferable products due to financial constraints.

Similar challenges were identified in a study with students in Palestine, where participants were compelled to use suboptimal options such as toilet paper or reusable underwear [50]. A systematic review encompassing studies on university populations in lower-income countries affirmed that financial constraints influenced the choice of menstrual hygiene products, often leading to the use of homemade sanitary towels [15].

In 2020, the European Parliament highlighted that 1 in 10 girls could not afford female hygiene products, advocating for measures to reduce taxes on these items across countries [51].

The literature demonstrates how menstrual inequalities can lead to detrimental outcomes for the physical and mental health of adolescents. It is estimated that the lack of access to menstrual products causes health issues for nearly 8% of high school students [52].

The cost of these products has come under increasing scrutiny, as they are rarely taxed as essential goods, and some menstruating individuals cannot afford them [53].

Addressing the issue in Spain, Organic Law No. 1/2023, enacted on 28 February, amended Organic Law No. 2/2010 of 3 March pertaining to sexual and reproductive health and voluntary pregnancy interruption. This legislation ensures free access to menstrual management products in educational centers, prisons, and social centers, particularly to aid women in vulnerable situations [6].

Our study has several important strengths, as it contributes knowledge regarding the perceptions and experiences of Spanish young women regarding menstruation. One limitation of the study is noted: all participants were predominantly university students, primarily from health sciences, which may imply a perceived higher level of knowledge. However, the participants’ actual level of knowledge was not objectively assessed, nor was their baseline level known. Additionally, the way in which questions about economic difficulties in accessing menstrual products may have influenced responses, as participants may have felt embarrassed, should be acknowledged. In this regard, it is important to mention that the socioeconomic level of the population has not been taken into account, and this could have biased, for example, the discourse on access to menstrual products. Furthermore, the use of contraception by the participants was not addressed. These aspects will be considered in future research.

## 5. Conclusions

By analyzing the perceptions of young Spanish women regarding aspects such as menstrual pain, the use of menstrual products, knowledge about menstruation, and menstrual poverty, our study provides crucial practical insights for further progress in this field. Regarding menstrual pain (dysmenorrhea), we noted its high prevalence and normalization by most participants, despite its potential debilitating effects on some occasions. We recommend implementing educational strategies to improve understanding of dysmenorrhea, emphasizing the importance of consulting healthcare professionals for assessment and guidance on appropriate management measures. This aims to discourage self-medication and its associated risks, while empowering young women to seek help without feeling ashamed.

In terms of menstrual products, our findings highlight the predominant use of pads and tampons, although there is a growing interest among young women in reusable menstrual products. It is crucial to develop educational interventions addressing practical aspects of using and managing these reusable products. Practical workshops allowing young women to familiarize themselves with the mechanisms of action and absorption capacity and enabling them to find the most suitable menstrual product for their needs are essential. Implementing simulation-based learning strategies can effectively integrate theory with practice.

Encouraging the proper and safe management of menstrual products by young women can help reduce negative experiences associated with menstruation. Exploring and promoting the accessibility of knowledge about reusable menstrual products, and incorporating them into digital content on menstrual hygiene, would be beneficial.

Although participants did not report experiencing menstrual poverty, they are aware of the significant economic burden it poses, considering the strong link between product prices and quality. They also understand how this impacts the experience of menstruation and its stigma. Therefore, it would be necessary to classify menstrual products as essential and reduce taxes on them.

Finally, adopting a comprehensive approach to menstrual health, contextualizing it as a health and human rights issue from before menarche to after menopause with physical, biological, and social implications, is crucial. This involves providing access to education, information, and scientific research, especially regarding conditions such as endometriosis and polycystic ovaries, to ensure that menstrual health is considered an integral part of the right to health, rather than just a hygiene issue. Dispelling misconceptions and myths that persist in society is also essential.

## Figures and Tables

**Table 1:** Categories and script of questions for focus groups.

Categories	Questions
Pain during menstruation	How would you describe your menstrual experiences in terms of pain? Have you ever consulted a specialist due to menstrual pain?
Menstrual products	What menstrual products do you use?
Menstrual management	Have you ever faced economic difficulties in accessing menstrual products?
Perceived knowledge about menstruation	Are you familiar with the menstrual cycle? Do you know the characteristics of a normal menstruation? What level of general knowledge do you believe you have about menstruation and the menstrual cycle? Would you like to receive more information on this topic? If so, what specific aspects are you interested in?

**Table 2:** Participants from discussion.

	G1	G2	G3	G4	Total	
N participants	9	11	17	8	45	
Age (SD)		20 (±1.52)	19.63 (±1.36)	20.64 (±0.47)	20.12 (±0.60)	20.23 (±1.18)
Context	Rural		5	11	6	48.88%
	Urban	9	6	6	2	51.11%
Occupation	Student	9	10	17	8	97.77%
	Worked	1	1			2.22%
Studies	Medicine	2				4.44%
	Nursing	7	11	17	4	86.66%
	Aerospace Engineering				3	6.66%
	Biology				1	2.22%

## Data Availability

Data are contained within the article.
